# Unraveling the after-hours dilemma: Consequences of overworking among teleworkers—A scoping review protocol

**DOI:** 10.1371/journal.pone.0330594

**Published:** 2025-08-28

**Authors:** Bao-Zhu Stephanie Long, Kishana Balakrishnar, Luke A. Fiorini, Aaron Howe, Ali Bani-Fatemi, Basem Gohar, Behdin Nowrouzi-Kia

**Affiliations:** 1 Department of Occupational Science and Occupational Therapy, Temerty Faculty of Medicine, University of Toronto, Toronto, Ontario, Canada; 2 Centre for Labour Studies, University of Malta, Msida, Malta; 3 Department of Population Medicine, University of Guelph, Guelph, Ontario, Canada; 4 Centre for Research in Occupational Safety & Health, Laurentian University, Sudbury, Ontario, Canada; 5 Krembil Research Institute, University Health Network, Toronto, Ontario, Canada; 6 Centre for Addiction and Mental Health, Toronto, Ontario, Canada; Instituto Federal Goiano, BRAZIL

## Abstract

**Background:**

Telework, also referred to as telecommuting, remote work, flexible work, and virtual work, involves working from a location different from the traditional office and often uses online communication technologies. Despite the numerous advantages associated with teleworking, it also raises concerns about work-life balance and health implications due to working after hours (WAH).

**Objective:**

This proposed study aims to understand the health consequences of teleworkers working beyond their scheduled hours.

**Methods:**

This review will search seven online databases (APA PsycINFO, Medline, Embase, Scopus, Business Source Premier, CINAHL, and Sociological Abstracts) to gather relevant articles. The inclusion criteria will encompass peer-reviewed studies published from 2010 onwards, focusing on WAH among teleworkers and reporting mental and physical health consequences. The exclusion criteria will include non-peer-reviewed articles, grey literature, and studies involving patients with pre-existing conditions.

**Discussion:**

This review will provide valuable insights into the mental and physical health consequences of WAH among teleworkers, underscoring the urgent need for strategies to mitigate these risks and promote overall well-being. Future efforts, including collaborations between researchers, industry leaders, and policymakers, can guide the development of targeted interventions and evidence-based policies that improve telework environments and support long-term worker health and productivity.

## Introduction

Telework, also known as “telecommuting”, “remote work”, “flexible work”, and “virtual work” [1], involves working from a location other than a traditional office setting, such as from home or a remote environment [2]. This practice utilizes online communication technologies to interact with colleagues, supervisors, customers, and staff members within and outside the organization to complete work-related tasks [2]. Telework was first introduced in the 1970s to reduce road congestion and conserve energy [3], which has now gained significant importance due to technological advancements [4] and its widespread adoption during the COVID-19 pandemic [5]. During the pandemic, telework enabled businesses to continue operating, thousands of workers to maintain their jobs, and the public sector to carry on delivering necessities [6]. The definition of teleworking has evolved from work done solely from home to include work performed virtually from various locations [7,8].

Teleworking offers several advantages, including shorter commutes, more flexible work schedules, increased leisure time with family, and decreased stress levels [9]. Reduced commuting time has been linked to decreased commuting stress and quality of life [10]. Teleworkers also benefit from managing personal and family obligations, such as caring for a sick child, without significantly disrupting work responsibilities [2]. Research suggests teleworking provides greater job autonomy for workers [11,12] with job tasks, workplace environment, and scheduling flexibility [2,13]. Telework has also been associated with increased worker productivity [14]. Statistics Canada reports that 90% of new teleworkers were at least as productive as they were in their usual work setting [15]. Similarly, the University of Chicago found remote work productivity to be 7% higher on average compared to in-office productivity [16]. However, this increase in productivity is contingent upon appropriate telework hours [17,18].

Working after hours (WAH), also referred to as “working outside agreed hours” [19], “long working hours” [20–22], “after-hours” [2,23], and many other extended-hour related terms, have become more common as telework increased during the COVID-19 pandemic [19,24]. Furthermore, WAH is known for working during non-working hours, including holidays, days off, and unpaid hours after work [19]. The rates of working after hours with telework has increased steadily since the COVID-19 pandemic due to increased workloads, greater expectations of after-hours availability between workers and supervisors, lack of boundaries between work and non-work roles at home, and increased strain on work-life balance of work, and familial and caregiver roles, especially for women [25,26]. The blurring of work-home boundaries when working from home can lead to a reluctance to disconnect from work after hours, hindering the ability to fulfill home responsibilities [25]. Many teleworkers also struggle to disconnect from work due to constant communication across group chats, email, or phone calls and remote meetings that extend beyond scheduled work hours [19]. These antecedents to WAH in teleworking jobs have been shown to have a significant relationship with burnout, increased work-related stress, poor mental health outcomes, and counterproductive work behaviors and attitudes towards their organization [26]. However, the literature has yet to address the characteristics of WAH that influence these work and health outcomes.

Several studies have documented the impacts of long work hours on teleworkers’ mental health and job satisfaction. A study conducted by Palma-Vasquez et al. [27] involving 278 classroom teachers in Chile working remotely, found that those who worked two or more hours of unpaid overtime were 2.89 times more likely to experience poor mental health outcomes compared to those who did not. Yeves et al. [28] also discovered that work overload, associated with telework, negatively impacted workers’ mental health due to burnout. Other studies highlight that work overload contributed to teleworkers’ strain [29] and emotional exhaustion [30,31]. A 2020 survey of EU workers working from home reported that those working long hours experienced feelings of isolation and emotional drainage, with 20% of employees working 41–60 hours per week feeling isolated and 39% feeling emotionally drained [32]. This contrasts with lower percentages of those working standard hours. Long working hours exacerbate mental health issues among teleworkers, contributing to increased loneliness and social isolation [33]. These mental health concerns may also exacerbate physical health conditions and risk of ergonomic injuries.

WAH is a significant occupational risk to physical health [24]. In 2016, working long hours led to over 745,000 people dying due to heart disease [34]. The WHO/ILO Joint Estimates indicate that the number of people who WAH has increased since 2000 [34]. Physical strain in overworked individuals can begin with acute physical reactions such as fatigue, insomnia, and undesirable lifestyle choices in response to stress [24].

Teleworkers, who rely on technological devices to complete tasks, may experience musculoskeletal issues from prolonged stationary positions and repetitive motions [35]. Increased WAH is associated with higher frequencies of upper and lower limb discomfort [36], back, neck, and shoulder pain [37], as well as a greater risk of hypertension [38,39], cardiovascular disease [24,40], stroke, and coronary heart disease [41,42]. Although ergonomic training and suitable workstations may reduce these risks [43], the lack of physical contact in teleworking may result in fewer breaks and extended work hours, increasing the probability of acquiring musculoskeletal issues [2].

Despite the mental and physical health burdens on teleworkers, research focusing on WAH as a primary issue, especially since the COVID-19 pandemic, is limited. There are a few studies that have examined the impact of WAH on musculoskeletal symptoms [36,44,45], the correlation between telework and worker well-being as a secondary outcome [2,46], and one study related to work hours on physical or mental well-being [41]. For instance, a cross-sectional study by Lee et al. [36], specifically examines the relationship between long working hours and work-related musculoskeletal symptoms, while the study by Krishnan et al. [44], also cross-sectional, focuses on the prevalence of work-related musculoskeletal disorders and their association with longer working hours and musculoskeletal pain. These studies, however, address only one consequence of after-hours work in detail, rather than both the mental and physical health outcomes of working beyond regular hours, as this protocol aims to do.

A meta-analysis conducted by Amiri [45], does examine broader impacts but still limits its focus to physical health outcomes. Furthermore, the study conducted by Beckel & Fisher [2] discusses telework and worker health, but only one section is dedicated to after-hours work, so WAH is not the primary outcome of this study, in contrast to the focus of our protocol. Similarly, a study by Furuya et al. [46] briefly touches on the well-being and health impacts of telework on workers, mentioning WAH in just one sentence. Finally, while the study by Ganster et al. [41] addresses the effects of WAH on physical or mental well-being, it was published in 2016. With the growing prevalence of telework [47–49], particularly since the COVID-19 pandemic, there is a pressing need for an updated, comprehensive review. Therefore, this scoping review protocol offers a timely update by focusing on both the mental and physical health consequences of WAH, a topic that warrants deeper examination. As the first step in the process, this protocol outlines the methodology and framework for conducting the full scoping review.

This review will comprehensively evaluate the effects associated with WAH among teleworkers. The primary objective(s) of this review will be to explore the prevalence and duration of WAH among teleworkers and to assess the effects it has on their work and well-being. To our knowledge, this study will be the first to comprehensively scope existing literature on the impacts of WAH while also considering WAH as a primary outcome. Given the substantial demands associated with WAH, often requiring employees to remain continuously available and engaged in work, we anticipate that the literature will reveal a consistent pattern of physical and mental health challenges associated with this work. The results gathered from this review will identify and map the available evidence on how WAH influences health and work outcomes to inform evidence-based strategies to improve telework practices and improve WAH telework policies within demand work professions.

## Materials and methods

### Study design and registration

A scoping review will be conducted to synthesize existing research on the consequences of WAH among teleworkers. Since aspects of the methodology and analysis may require refinement as the review progresses, we acknowledge the following assumptions: (1) Existing literature sufficiently examines after-hours work among teleworkers to allow for meaningful and comprehensive synthesis, and (2) studies will provide relevant data on the health impacts associated with after-hours work. Moreover, certain aspects of our analysis are exploratory. Given the growing nature of telework and the variability in how WAH is defined across the literature, we expect identifying emerging themes beyond the predefined categories. This explanatory approach will allow for an in-depth understanding of the consequences of WAH among teleworkers. From our understanding, no knowledge syntheses have investigated the mental and physical impacts of after-hours work in the context of telework as a primary outcome in recent years. The Preferred Reporting Items for Systematic review and Meta-analysis Protocols (PRISMA-P) was considered when developing this protocol [50]. The forthcoming scoping review will be prepared using the guidelines outlined by the Joanna Briggs Institute (JBI) to ensure the rigor and replicability of our study [51]. This protocol is registered with the Open Science Framework under https://doi.org/10.17605/OSF.IO/CR3XP.

### Search strategy

This scoping review will search seven databases for existing articles on the impact of WAH on the mental and physical health of teleworkers: APA PsycINFO (Ovid), Medline (Ovid), Embase (Ovid), Scopus (Elsevier), Business Source Premier (Ebsco), CINAHL (Ebsco), and Sociological Abstracts (ProQuest). These databases were selected for their broad coverage of healthcare and professional sectors, allowing for a comprehensive examination of the topic from multiple perspectives. This selection was made in consultation with a librarian whose expertise in database selection ensures the inclusion of the most relevant and effective resources. To ensure relevance to current technologies and teleworking practices, studies must be published from 2010 onwards. Sources will be stored within Zotero [52], a reference management program.

Authors B-ZSL and LAF created the search syntax for all databases. The search included the use of synonyms associated with “teleworkers” (patient/population), combined with terms related to “working after hours” (exposure/situation), and “health consequences” (outcome). This was done in collaboration with the University of Toronto Scarborough Health and Society Librarian and the research team, with expertise in occupational health and therapy, workplace mental and physical health, and lived experiences in remote working environments. It is recommended that researchers and readers reference the Supporting Information for a comprehensive overview of the search methodology. The search strategy considered the changing nature of telework, particularly in the wake of COVID-19, and focused on studies published between 2010 and the present. Only English-Language articles will be included. Study eligibility criteria are presented in Tables 1 and 2 below.

**Table 1:** Inclusion criteria.

	Inclusion Criteria
**1**	The primary outcome of the study must be listed as “working after hours” or a relative synonym of WAH (e.g., working outside agreed hours) (refer to the Supporting Information for a full list of allied terms).
**2**	Research must describe how WAH leads to mental and physical health consequences among teleworkers specifically.
**3**	All research articles should be peer-reviewed.
**4**	Papers must include individuals of working age (i.e., 18+).
**5**	WAH studies should be conducted in environments that involve telecommuting, telework, virtual work, remote work, or home-based office setups.
**6**	Research papers must be written in English or have English translations available for publishing in the journal.
**7**	Research will encompass all regions to document a diverse range of remote work settings.
**8**	Studies must focus exclusively on workers who perform their job entirely remotely or as teleworkers, without any in-person or on-site work requirements.

### Inclusion and exclusion criteria

**Table 2:** Exclusion criteria.

	Exclusion Criteria
**1**	Any unpublished research, opinion pieces, book chapters, conference papers and abstracts, review articles, knowledge syntheses, commentaries, or grey literature.
**2**	Any research articles whereby teleworkers return-to-work remotely due to a work-related injury or illness.

### Data collection

Two independent reviewers (B-ZSL, KB) will use Covidence [53], a publicly accessible system for managing reviews, to organize each research article found in the literature search, adhering to the search method described in the Supporting Information. Covidence’s algorithmic approach will eliminate duplicate entries, and one reviewer will manually verify the remaining entries. Following that, each title and abstract will be individually assessed by both reviewers, who will then eliminate any papers that fail to meet the predetermined inclusion requirements. The remaining studies that meet the specified requirements will have their full texts thoroughly analyzed and their relevance evaluated by the two reviewers. A third reviewer (BNK) will ensure a comprehensive and uniform screening and selection procedure. The research team will be involved when necessary to discuss disagreements between reviewers until an agreement is established. After the screening procedure, Covidence will populate the PRISMA flow diagram for scoping reviews, showing which articles qualified through the title and abstract screening and which ones did not. It will also include the full-text screening results and the reasoning behind excluding certain articles.

### Data extraction

Two independent reviewers (B-ZSL and KB) will extract data. Pilot testing will be performed in advance to ensure inter-rater agreement among the reviewers. The data will be charted in Microsoft Excel [54] using the JBI template encompassing the following categories: (1) Title of the study; (2) author name(s); (3) publication year; (4) country of origin; (5) study design; (6) measurement scale (own scale or standardized scale); (7) study participant characteristics (e.g., age, gender, occupation, socioeconomic status, number of household members); (8) study objectives; (9) timing and/or duration of after-hours work; (10) type of work completed while WAH; (11) physical environment of work station when teleworking (12) presence of specific telework policies; (13) health consequences (e.g., mental health, physical health, ergonomic factors, job satisfaction, and work productivity) of WAH among all types of teleworkers. The categories from the data extraction table will aid in formulating potential themes for the scoping review. Since data collection and entry are iterative processes in scoping reviews, the data extraction table will be continuously updated when necessary. The full scoping review will document all modifications made.

### Data synthesis

Using a Microsoft Excel spreadsheet, the two independent reviewers will cross-check their results and conclusions after extracting the data and then combine their data charts. Following this procedure, the reviewers will have discussions in which they will determine the major themes they came across when reading the literature presented in this review. The findings will be conceptualized using a socioecological framework. This stage will identify any conflicts and communicate them to a third-party member (BNK) for resolution.

### Critical appraisal

A thorough critical appraisal will be performed by the two independent reviewers for all included studies. The Agency for Healthcare Research and Quality (AHRQ) tool will be used for cross-sectional studies [55], while the Newcastle-Ottawa Scale (NOS) will be applied for cohort and case-control studies [56]. Moreover, for randomized controlled trials, qualitative studies, diagnostic studies, and economic evaluations, we will utilize the Critical Appraisal Skills Programme (CASP) tool [57]. Each reviewer will evaluate the studies independently for any risk of bias by categorizing them as either low, moderate, or high. Any conflict between the reviewers will be resolved through discussion. Any matter will be escalated to the rest of the research team if an agreement cannot be reached. The results of the appraisal will be recorded separately.

### Dissemination

With the publication of this protocol, the research team at the University of Toronto will conduct the scoping review following the procedures outlined. The final manuscript will be prepared and submitted to a peer-reviewed journal supervised by the senior author (BNK).

## Discussion

This proposed scoping review will aim to understand the existing literature on WAH and investigate how this practice contributes to mental and physical health consequences. We believe that this review will enhance knowledge of mental factors such as burnout, stress, anxiety, depression, presenteeism, absenteeism, and physical factors like musculoskeletal disorders (MSDs), strain or injuries on physical anatomy, and various physiological illnesses relative to the prevalence of WAH. By reviewing the existing literature, this review will clarify the intricate relationship between prolonged work hours and health outcomes, providing a foundation for initiatives to encourage healthier work habits and improve the well-being of teleworking populations.

WAH, characterized by spending additional time on work-related activities beyond the expected working schedule [58], is prevalent among teleworkers due to workplace pressure experienced by teleworkers, who describe feeling overburdened by the volume of work they must complete, having to focus on too many projects at once, and reporting that the demands of their jobs outweigh the amount of time they have to complete the work [59]. These challenges can lead to both mental and physical strain that might result from acute physiological reactions such as exhaustion, stress, disturbed sleep, and unhealthful lifestyle adjustments brought on by the stress [24], to increased time and energy demands, which are connected to distress and burnout [60]. Furthermore, WAH lowers productivity and increases the risk of disease and occupational risks [24]. These factors are all vital to understanding how to safeguard the well-being of teleworkers, ensuring they can perform their duties effectively and safely. Employers must prioritize strategies that promote a balanced work environment to support the health and performance of their teleworking staff.

A study by Heiden et al. [61] discovered employees who teleworked more than their preferred hours experienced poorer mental well-being than those who teleworked less than their ideal amount. This underscores the importance of the preferred and actual extent of telework on workers’ well-being. Another study reported that employees who teleworked eight hours or less were less likely to report depressive symptoms [62]. In fact, telework, in general, is associated with negative mental health outcomes [63]. Employees working from home experienced increased rates of depression and anxiety [64]. Additionally, compared to employees working in-person, teleworkers endure greater emotions of loneliness, irritability, worry, and guilt which lead them to experience negative mental health outcomes [33]. Decreased physical activity, increased screen time, and greater snacking were shown to be correlated with a higher incidence of depression, loneliness, and anxiety [65]. With teleworking leading to several mental health consequences, working extensive periods within this environment can potentially exacerbate these symptoms and outcomes.

Employees who telework for extended periods are more likely to suffer from MSDs and experience body strains. A study conducted by Gosain et al. [66] indicated a greater prevalence of neck pain observed among home workers who stay in one posture and front of a screen for long hours. Additionally, Gupta et al. [67] stated that participants teleworking for long hours exhibited an increased incidence of MSDs as their job required them to sit for extended periods. Telework is associated with less mobility and requirement for standing up, increasing sedentary activity [68]. Moreover, with home-based work, healthy modes of commuting, such as walking or cycling, have decreased [69]. Several studies have established the relationship between musculoskeletal pain among teleworkers and physical inactivity and sedimentary behaviour [66,67,70]. Likewise, working for long hours at home may aggravate musculoskeletal (MSK) pain, considering how the use of mobile devices by teleworkers, such as laptops, phones, and tablets, are more likely to lead to poor posture than desktop computers [36,71,72]. In addition to MSK disorders and strain, working long hours at home may lead to developing severe diseases. Evidence indicates that individuals working 55 hours or more per week have an increased likelihood of cardiovascular conditions compared to those who work 35–40 hours a week, due to exposure of increased stress levels [73]. Additionally, longer working hours at home result in poorer sleep quality as they have reduced exposure to sunlight, which is crucial for helping maintain regular circadian rhythms [74], further contributing to an increased risk of cardiovascular disease [75].

Working after hours is strongly influenced by various demographic factors which renders certain populations more susceptible to health implications of telework [76–79]. Research indicates that younger workers are more likely than older colleagues to engage in after-hours work, experiencing heightened levels of work-like conflict in return [78,79]. Additionally, a study by Senturk et al. [76] observed that remote-working women in Turkey were more likely to work beyond regular hours, primarily due to dual pressures of increased workloads and household responsibilities. The challenges faced by women teleworkers are further compounded by traditional gender roles, which often impose additional expectations regarding household and family care duties [80]. As a result, women are particularly vulnerable to work-family conflict and associated mental health challenges during telecommuting [81]. Moreover, socioeconomic status plays a significant role in after-hours work, with workers from lower socioeconomic backgrounds often experiencing greater pressure to remain available outside standard work hours, thus leading to more negative impacts on well-being [77]. Although the demographic factors of age, gender, and socioeconomic status are linked to WAH, more research needs to be done to explore how these individual differences contribute to the poor mental and physical health consequences derived from WAH.

Despite the association between teleworking and after-hours work [59], which often leads to various health implications, many workers perceive benefits [24], such as time and cost savings [82]. Hybrid work arrangements present an opportunity to balance these advantages with the potential drawbacks of after-hours work. Research has shown that hybrid work can positively influence work-life balance [83–85]. A study conducted by Nugroho and Desiana [86] found that hybrid working arrangements directly contribute to well-being by enhancing work-life balance and increasing employee satisfaction. This suggests that hybrid work could provide a promising solution to alleviate the negative aspects of telework while also maintaining some of its benefits.

The study’s methodology has a notable limitation. The focus on peer-reviewed research published in English or with English translations may introduce language and publication bias, potentially excluding valuable non-English studies and limiting the diversity of perspectives and findings. In the case that non-English articles are translated, this could also introduce additional biases, as translation is not always a precise or neutral process. Errors in translation, or even the selection of certain words or phrases, may inadvertently change the meaning or context of key findings. This could falsify the original study’s conclusions, resulting in inaccurate or misleading interpretations. We anticipate that this review will serve as the first step in addressing the gaps in current research and policy regarding the prevalence and effects of WAH.

### Review registration

The protocol has been formally registered with the Open Science under https://doi.org/10.17605/OSF.IO/CR3XP.

## Supporting information

S1 File PRISMA-P-checklist. (DOCX)

S2 File Search strategy. (DOCX)
