# Supplementary material for: Unraveling the after-hours dilemma: Consequences of overworking among teleworkers—A scoping review protocol
Source: PLoS One. 2025 Aug 28;20(8):e0330594. doi: 10.1371/journal.pone.0330594 (PMC12393701; doi:10.1371/journal.pone.0330594)
Supplement: S2 File — (DOCX) [file pone.0330594.s002.docx]

***APA PsycINFO (Ovid)***

1 telecommuting/ or teleworkers/ or virtual teams/

2 telework*.mp.

3 telecommut*.mp.

4 (telework* adj3 (professional* or employ* or job or staff*)).mp.

5 (virtual* adj3 (professional* or work* or employ* or job or staff*)).mp.

6 (remote* adj3 (professional* or work* or employ* or job or staff*)).mp.

7 (off-site adj3 (professional* or work* or employ* or job or staff*)).mp.

8 (home-based adj3 (professional* or work* or employ* or job or staff* or telework*)).mp.

9 (asynchronous adj3 (professional* or work* or employ* or job or staff* or telework*)).mp.

10 home-work*.mp.

11 work* from home.mp.

12 work* remotely.mp.

13 (e-work* or ework*).mp.

14 flexible work*.mp.

15 hybrid work*.mp.

16 1 or 2 or 3 or 4 or 5 or 6 or 7 or 8 or 9 or 10 or 11 or 12 or 13 or 14 or 15

17 workaholism/ or occupational stress/ or "work (attitudes toward)"/ or work rest cycles/

18 work-life balance/ or work load/ or work scheduling/

19 (work* adj3 (after hour* or beyond hour* or extended hour* or outside agreed hour* or past schedule* or outside normal hour*)).mp.

20 long* work* hour*.mp.

21 irregular hour*.mp.

22 beyond work* hour*.mp.

23 leaveism*.mp.

24 (extended adj3 (period* or hour* or shift* or schedule* or work schedule*)).mp.

25 night work*.mp.

26 weekend work*.mp.

27 ((informal or work*) adj3 overtime).mp.

28 work* late.mp.

29 work* off the clock.mp.

30 stay* late.mp.

31 overwork*.mp.

32 17 or 18 or 19 or 20 or 21 or 22 or 23 or 24 or 25 or 26 or 27 or 28 or 29 or 30 or 31

33 fatigue/ or burnout/ or chronic fatigue syndrome/ or compassion fatigue/ or digital fatigue/ or emotional exhaustion/

34 ((work* or job or job-related or occupation* or physical or psychological or mental or emotional) adj3 fatigue).mp.

35 ((work* or job or job-related or occupation* or physical or psychological or mental or emotional) adj3 burnout).mp.

36 ((work* or job or job-related or occupation* or physical or psychological or mental or emotional) adj3 exhaustion).mp.

37 ((work* or job or job-related or occupation* or physical or psychological or mental or emotional) adj3 tension).mp.

38 stress/ or acute stress/ or chronic stress/ or environmental stress/ or occupational stress/ or perceived stress/ or physiological stress/ or posttraumatic stress/ or psychological stress/ or social stress/ or stress reactions/

39 employee well being/ or job demands/ or job resources/ or "quality of work life"/ or work related illnesses/

40 ((work* or job or job-related or occupation* or physical or psychological or mental or emotional) adj3 stress).mp.

41 anxiety/ or health anxiety/ or performance anxiety/ or social anxiety/

42 ((work* or job or job-related or occupation* or physical or psychological or mental or emotional) adj3 anxiety).mp.

43 anxiety disorders/ or generalized anxiety disorder/ or panic attack/ or panic disorder/ or separation anxiety disorder/ or anxiety management/ or illness anxiety disorder/ or obsessive compulsive disorder/

44 mental disorders/

45 (mental adj3 (disorder* or illness* or fatigue)).mp.

46 ((reduced or declining or deteriorating or mental or physical or psychological or emotional or occupation* or work-related or work*) adj3 health).mp.

47 ((diminished or job-related or job or work* or work-related or mental or physical or psychological or occupation* or emotional) adj3 wellness).mp.

48 job satisfaction/ or employee attitudes/ or career change/ or employee engagement/ or employee retention/ or employee well being/ or role satisfaction/

49 ((diminished or reduced or deteriorating or declining or job-related or job or work* or work-related or mental or physical or psychological or occupation* or emotional) adj3 well-being).mp.

50 exp Musculoskeletal Disorders/

51 (musculoskeletal adj3 (disorder* or condition* or issue* or disease* or pain)).mp.

52 pain/ or acute pain/ or back pain/ or chronic pain/ or headache/ or myofascial pain/ or neuralgia/ or neuropathic pain/ or pain management/ or physical disorders/

53 ((physical or somatic or bodily) adj3 pain).mp.

54 ((physical or bodily or somatic) adj3 discomfort).mp.

55 ((physical or bodily or somatic) adj3 distress).mp.

56 reduced performance.mp.

57 diminished productivity.mp.

58 demotivation.mp.

59 disengagement.mp.

60 disinterest.mp.

61 lack of motivation.mp.

62 change of job.mp.

63 employment change*.mp.

64 job transition*.mp.

65 career shift*.mp.

66 family conflict/ or marital conflict/ or domestic violence/

67 ((family or co-worker or coworker or work* or workplace or interpersonal or peer) adj3 conflict*).mp.

68 employee absenteeism/

69 (absenteeism or non-attendance or absence from work* or skipping work* or missing workday*).mp.

70 illness behavior/ or "physical illness (attitudes toward)"/

71 (presenteeism or attending work while ill or work* while sick or on-the-job-sickness or working despite illness).mp.

72 (poor health behavio#r* or unhealthy habit* or negative health practice*).mp.

73 33 or 34 or 35 or 36 or 37 or 38 or 39 or 40 or 41 or 42 or 43 or 44 or 45 or 46 or 47 or 48 or 49 or 50 or 51 or 52 or 53 or 54 or 55 or 56 or 57 or 58 or 59 or 60 or 61 or 62 or 63 or 64 or 65 or 66 or 67 or 68 or 69 or 70 or 71 or 72

74 16 and 32 and 73

75 limit 74 to (english language and yr="2010 -Current")

***MEDLINE (Ovid)***

1 exp Teleworking/

2 telework*.tw,kf.

3 telecommut*.tw,kf.

4 (telework* adj3 (professional* or employ* or job or staff*)).tw,kf.

5 (virtual* adj3 (professional* or work* or employ* or job or staff*)).tw,kf.

6 (remote* adj3 (professional* or work* or employ* or job or staff*)).tw,kf.

7 (off-site adj3 (professional* or work* or employ* or job or staff*)).tw,kf.

8 (home-based adj3 (professional* or work* or employ* or job or staff* or telework*)).tw,kf.

9 home-work*.tw,kf.

10 work* from home.tw,kf.

11 (asynchronous adj3 (professional* or work* or employ* or job or staff* or telework*)).tw,kf.

12 work* remotely.tw,kf.

13 (e-work* or ework*).tw,kf.

14 flexible work*.tw,kf.

15 hybrid work*.tw,kf.

16 1 or 2 or 3 or 4 or 5 or 6 or 7 or 8 or 9 or 10 or 11 or 12 or 13 or 14 or 15

17 Work-Life Balance/

18 work-life balance.tw,kf.

19 overtime work*.tw,kf.

20 workaholism.tw,kf.

21 long* work* hour*.tw,kf.

22 irregular hour*.tw,kf.

23 beyond work* hour*.tw,kf.

24 beyond hour*.tw,kf.

25 leaveism*.tw,kf.

26 (extended adj3 (period* or hour* or shift* or schedule* or work schedule*)).tw,kf.

27 night work*.tw,kf.

28 weekend work*.tw,kf.

29 ((informal or work*) adj3 overtime).tw,kf.

30 work* late.tw,kf.

31 work* off the clock.tw,kf.

32 stay* late.tw,kf.

33 overwork*.tw,kf.

34 overtime*.tw,kf.

35 17 or 18 or 19 or 20 or 21 or 22 or 23 or 24 or 25 or 26 or 27 or 28 or 29 or 30 or 31 or 32 or 33 or 34

36 fatigue/ or emotional exhaustion/ or mental fatigue/ or overtraining syndrome/

37 ((work* or job or job-related or occupation* or physical or psychological or mental or emotional) adj3 fatigue).tw,kf.

38 Burnout, Psychological/ or Burnout, Professional/

39 ((work* or job or job-related or occupation* or physical or psychological or mental or emotional) adj3 burnout).tw,kf.

40 emotional exhaustion/

41 ((work* or job or job-related or occupation* or physical or psychological or mental or emotional) adj3 exhaustion).tw,kf.

42 ((work* or job or job-related or occupation* or physical or psychological or mental or emotional) adj3 tension).tw,kf.

43 Occupational Stress/

44 stress, psychological/ or time pressure/

45 Subjective Stress/ or Adaptation, Psychological/

46 ((work* or job or job-related or occupation* or physical or psychological or mental or emotional) adj3 stress).tw,kf.

47 Anxiety/ or Anxiety, Separation/ or exp Anxiety Disorders/ or Performance Anxiety/

48 ((work* or job or job-related or occupation* or physical or psychological or mental or emotional) adj3 anxiety).tw,kf.

49 Mental Disorders/

50 (mental adj3 (disorder* or illness* or fatigue)).tw,kf.

51 ((reduced or declining or deteriorating or mental or physical or psychological or emotional or occupation* or work-related or work*) adj3 health).tw,kf.

52 ((diminished or job-related or job or work* or work-related or mental or physical or psychological or occupation* or emotional) adj3 wellness).tw,kf.

53 Job Satisfaction/

54 ((diminished or reduced or deteriorating or declining or job-related or job or work* or work-related or mental or physical or psychological or occupation* or emotional) adj3 well-being).tw,kf.

55 exp Musculoskeletal Diseases/

56 (musculoskeletal adj3 (disorder* or condition* or issue* or disease* or pain)).tw,kf.

57 Chronic Pain/ or Back Pain/ or Low Back Pain/ or exp Pain/ or Neck Pain/ or Pain Management/ or Eye Pain/ or Acute Pain/ or Abdominal Pain/ or Musculoskeletal Pain/ or Shoulder Pain/ or Chest Pain/

58 ((physical or somatic or bodily) adj3 pain).tw,kf.

59 ((physical or bodily or somatic) adj3 discomfort).tw,kf.

60 ((physical or bodily or somatic) adj3 distress).tw,kf.

61 reduced performance.tw,kf.

62 diminished productivity.tw,kf.

63 demotivation.tw,kf.

64 disengagement.tw,kf.

65 disinterest.tw,kf.

66 lack of motivation.tw,kf.

67 change of job.tw,kf.

68 employment change*.tw,kf.

69 job transition*.tw,kf.

70 career shift*.tw,kf.

71 Family Conflict/

72 ((family or co-worker or coworker or work* or workplace or interpersonal or peer) adj3 conflict*).tw,kf.

73 exp Absenteeism/

74 (absenteeism or non-attendance or absence from work* or skipping work* or missing workday*).tw,kf.

75 exp Presenteeism/ or exp Sick Leave/

76 (presenteeism or attending work while ill or work* while sick or on-the-job-sickness or working despite illness).tw,kf.

77 (poor health behavio#r* or unhealthy habit* or negative health practice*).tw,kf.

78 36 or 37 or 38 or 39 or 40 or 41 or 42 or 43 or 44 or 45 or 46 or 47 or 48 or 49 or 50 or 51 or 52 or 53 or 54 or 55 or 56 or 57 or 58 or 59 or 60 or 61 or 62 or 63 or 64 or 65 or 66 or 67 or 68 or 69 or 70 or 71 or 72 or 73 or 74 or 75 or 76 or 77

79 16 and 35 and 78

80 limit 79 to (english language and yr="2010 -Current")

***Embase (Ovid)***

1 telecommuting/ or "work from home"/

2 telework*.tw,kf.

3 telecommut*.tw,kf.

4 (telework* adj3 (professional* or employ* or job or staff*)).tw,kf.

5 (virtual* adj3 (professional* or work* or employ* or job or staff*)).tw,kf.

6 (remote* adj3 (professional* or work* or employ* or job or staff*)).tw,kf.

7 (off-site adj3 (professional* or work* or employ* or job or staff*)).tw,kf.

8 (home-based adj3 (professional* or work* or employ* or job or staff* or telework*)).tw,kf.

9 home-work*.tw,kf.

10 work* from home.tw,kf.

11 (asynchronous adj3 (professional* or work* or employ* or job or staff* or telework*)).tw,kf.

12 work* remotely.tw,kf.

13 (e-work* or ework*).tw,kf.

14 flexible work*.tw,kf.

15 hybrid work*.tw,kf.

16 1 or 2 or 3 or 4 or 5 or 6 or 7 or 8 or 9 or 10 or 11 or 12 or 13 or 14 or 15

17 workaholism/ or behavioral addiction/

18 work-life balance/

19 (work* adj3 (after hour* or beyond hour* or extended hour* or outside agreed hour* or past schedule* or outside normal hour*)).tw,kf.

20 long* work* hour*.tw,kf.

21 irregular hour*.tw,kf.

22 beyond work* hour*.tw,kf.

23 leaveism*.tw,kf.

24 (extended adj3 (period* or hour* or shift* or schedule* or work schedule*)).tw,kf.

25 night work*.tw,kf.

26 weekend work*.tw,kf.

27 ((informal or work*) adj3 overtime).tw,kf.

28 work* late.tw,kf.

29 work* off the clock.tw,kf.

30 staying late.tw,kf.

31 overwork*.tw,kf.

32 17 or 18 or 19 or 20 or 21 or 22 or 23 or 24 or 25 or 26 or 27 or 28 or 29 or 30 or 31

33 fatigue/ or chronic fatigue syndrome/ or exhaustion/ or mental fatigue/ or overtraining syndrome/

34 alert fatigue/ or health personnel/ or compassion fatigue/

35 ((work* or job or job-related or occupation* or physical or psychological or mental or emotional) adj3 fatigue).tw,kf.

36 burnout/ or mental stress/ or professional burnout/

37 ((work* or job or job-related or occupation* or physical or psychological or mental or emotional) adj3 burnout).tw,kf.

38 ((work* or job or job-related or occupation* or physical or psychological or mental or emotional) adj3 exhaustion).tw,kf.

39 ((work* or job or job-related or occupation* or physical or psychological or mental or emotional) adj3 tension).tw,kf.

40 physiological stress/ or acute stress/ or chronic stress/ or physically induced stress/

41 job stress/ or role stress/

42 ((work* or job or job-related or occupation* or physical or psychological or mental or emotional) adj3 stress).tw,kf.

43 anxiety/ or performance anxiety/ or social anxiety/

44 ((work* or job or job-related or occupation* or physical or psychological or mental or emotional) adj3 anxiety).tw,kf.

45 anxiety disorder/ or acute stress disorder/ or anxiety neurosis/ or distress syndrome/ or generalized anxiety disorder/ or "mixed anxiety and depression"/ or obsessive compulsive disorder/

46 mental disease/

47 (mental adj3 (disorder* or illness* or fatigue)).tw,kf.

48 ((reduced or declining or deteriorating or mental or physical or psychological or emotional or occupation* or work-related or work*) adj3 health).tw,kf.

49 ((diminished or job-related or job or work* or work-related or mental or physical or psychological or occupation* or emotional) adj3 wellness).tw,kf.

50 job satisfaction/

51 ((diminished or reduced or deteriorating or declining or job-related or job or work* or work-related or mental or physical or psychological or occupation* or emotional) adj3 well-being).tw,kf.

52 exp musculoskeletal disease/

53 (musculoskeletal adj3 (disorder* or condition* or issue* or disease* or pain)).tw,kf.

54 pain/ or abdominal pain/ or bone pain/ or chronic pain/ or eye pain/ or eyelid pain/ or limb pain/

55 acute pain/ or back pain/ or chest pain/ or headache/ or musculoskeletal pain/ or neck pain/

56 ((physical or somatic or bodily) adj3 pain).tw,kf.

57 ((physical or bodily or somatic) adj3 discomfort).tw,kf.

58 ((physical or bodily or somatic) adj3 distress).tw,kf.

59 reduced performance.tw,kf.

60 diminished productivity.tw,kf.

61 demotivation.tw,kf.

62 disengagement.tw,kf.

63 disinterest.tw,kf.

64 lack of motivation.tw,kf.

65 change of job.tw,kf.

66 employment change*.tw,kf.

67 job satisfaction/

68 career mobility/

69 job transition*.tw,kf.

70 career shift*.tw,kf.

71 family conflict/

72 ((family or co-worker or coworker or work* or workplace or interpersonal or peer) adj3 conflict*).tw,kf.

73 Absenteeism/

74 (absenteeism or non-attendance or absence from work* or skipping work* or missing workday*).tw,kf.

75 Presenteeism/

76 (presenteeism or attending work while ill or work* while sick or on-the-job-sickness or working despite illness).tw,kf.

77 (poor health behavio#r* or unhealthy habit* or negative health practice*).tw,kf.

78 33 or 34 or 35 or 36 or 37 or 38 or 39 or 40 or 41 or 42 or 43 or 44 or 45 or 46 or 47 or 48 or 49 or 50 or 51 or 52 or 53 or 54 or 55 or 56 or 57 or 58 or 59 or 60 or 61 or 62 or 63 or 64 or 65 or 66 or 67 or 68 or 69 or 70 or 71 or 72 or 73 or 74 or 75 or 76 or 77

79 16 and 32 and 78

80 limit 79 to (english language and yr="2010 -Current")

***Scopus (Elsevier)***

1 TITLE-ABS-KEY ( telework* )
2 TITLE-ABS-KEY ( telecommut* )

3 TITLE-ABS-KEY ( ( telework* W/3 ( professional* OR employ* OR job OR staff* ) ) )

4 TITLE-ABS KEY ( ( virtual* W/3 ( professional* OR work* OR employ* OR job OR staff* ) ) )

5 TITLE-ABS-KEY ( ( remote* W/3 ( professional* OR work* OR employ* OR job OR staff* ) ) )

6 TITLE-ABS-KEY ( ( off-site W/3 ( professional* OR work* OR employ* OR job OR staff* ) ) )

7 TITLE-ABS-KEY ( ( home-based W/3 ( professional* OR work* OR employ* OR job OR staff* OR telework* ) ) )

8 TITLE-ABS-KEY ( ( asynchronous W/3 ( professional* OR work* OR employ* OR job OR staff* OR telework* ) ) )

9 TITLE-ABS-KEY ( home-work* )

10 TITLE-ABS-KEY ( "work* from home" )

11 TITLE-ABS-KEY ( "work* remotely" )

12 TITLE-ABS-KEY ( ( e-work* OR ework* ) )

13 TITLE-ABS-KEY ( "flexible work*" )

14 TITLE-ABS-KEY ( "hybrid work*" )

15 ( TITLE-ABS-KEY ( telework* ) ) OR ( TITLE-ABS-KEY ( telecommut* ) ) OR ( TITLE-ABS-KEY ( ( telework* W/3 ( professional* OR employ* OR job OR staff* ) ) ) ) OR ( TITLE-ABS-KEY ( ( virtual* W/3 ( professional* OR work* OR employ* OR job OR staff* ) ) ) ) OR ( TITLE-ABS-KEY ( ( remote* W/3 ( professional* OR work* OR employ* OR job OR staff* ) ) ) ) OR ( TITLE-ABS-KEY ( ( off-site W/3 ( professional* OR work* OR employ* OR job OR staff* ) ) ) ) OR ( TITLE-ABS-KEY ( ( home-based W/3 ( professional* OR work* OR employ* OR job OR staff* OR telework* ) ) ) ) OR ( TITLE-ABS-KEY ( ( asynchronous W/3 ( professional* OR work* OR employ* OR job OR staff* OR telework* ) ) ) ) OR ( TITLE-ABS-KEY ( home-work* ) ) OR ( TITLE-ABS-KEY ( "work* from home" ) ) OR ( TITLE-ABS-KEY ( "work* remotely" ) ) OR ( TITLE-ABS-KEY ( ( e-work* OR ework* ) ) ) OR ( TITLE-ABS-KEY ( "flexible work*" ) ) OR ( TITLE-ABS-KEY ( "hybrid work*" ) )

16 TITLE-ABS-KEY ( ( work* W/3 ( "after hour*" OR "beyond hour*" OR "extended hour*" OR "outside agreed hour*" OR "past schedule*" OR "outside normal hour*" ) )

17 TITLE-ABS-KEY ( "long* work* hour*" ) )

18 TITLE-ABS-KEY ( "irregular hour*" ) )

19 TITLE-ABS-KEY ( "beyond work* hour*" )

20 TITLE-ABS-KEY ( leaveism* )

21 TITLE-ABS KEY ( ( extended W/3 ( period* OR hour* OR shift* OR schedule* OR "work schedule*" ) ) )

22 TITLE-ABS-KEY ( "night work*" )

23 TITLE-ABS-KEY ( "weekend work*" )

24 TITLE-ABS-KEY ( ( ( informal OR work* ) W/3 overtime ) )

25 TITLE-ABS-KEY ( "work* late" )

26 TITLE-ABS-KEY ( "work* off the clock" )

27 TITLE-ABS-KEY ( "stay* late" )

28 TITLE-ABS-KEY ( overwork* )

29 TITLE-ABS-KEY ( workaholism )

30 TITLE-ABS-KEY ( "work-life balance" )

31 TITLE-ABS-KEY ( "work load" )

32 TITLE-ABS-KEY ( "work scheduling" )

33 TITLE-ABS-KEY ( "work rest cycle*" )

34 ( TITLE-ABS-KEY ( ( work* W/3 ( "after hour*" OR "beyond hour*" OR "extended hour*" OR "outside agreed hour*" OR "past schedule*" OR "outside normal hour*" ) ) ) ) OR ( TITLE-ABS-KEY ( "long* work* hour*" ) ) OR ( TITLE-ABS-KEY ( "irregular hour*" ) ) OR ( TITLE-ABS-KEY ( "beyond work* hour*" ) ) OR ( TITLE-ABS-KEY ( leaveism* ) ) OR ( TITLE-ABS-KEY ( ( extended W/3 ( period* OR hour* OR shift* OR schedule* OR "work schedule*" ) ) ) ) OR ( TITLE-ABS-KEY ( "night work*" ) ) OR ( TITLE-ABS-KEY ( "weekend work*" ) ) OR ( TITLE-ABS-KEY ( ( ( informal OR work* ) W/3 overtime ) ) ) OR ( TITLE-ABS-KEY ( "work* late" ) ) OR ( TITLE-ABS-KEY ( "work* off the clock" ) ) OR ( TITLE-ABS-KEY ( "stay* late" ) ) OR ( TITLE-ABS-KEY ( overwork* ) ) OR ( TITLE-ABS-KEY ( workaholism ) ) OR ( TITLE-ABS-KEY ( "work-life balance" ) ) OR ( TITLE-ABS-KEY ( "work load" ) ) OR ( TITLE-ABS-KEY ( "work scheduling" ) ) OR ( TITLE-ABS-KEY ( "work rest cycle*" ) )

35 TITLE-ABS-KEY ( ( ( work* OR job OR job-related OR occupation* OR physical OR psychological OR mental OR emotional ) W/3 fatigue ) )

36 TITLE-ABS-KEY ( ( ( work* OR job OR job-related OR occupation* OR physical OR psychological OR mental OR emotional ) W/3 burnout ) )

37 TITLE-ABS-KEY ( ( ( work* OR job OR job-related OR occupation* OR physical OR psychological OR mental OR emotional ) W/3 exhaustion ) )

38 TITLE-ABS-KEY ( ( ( work* OR job OR job-related OR occupation* OR physical OR psychological OR mental OR emotional ) W/3 tension ) )

39 TITLE-ABS-KEY ( ( ( work* OR job OR job-related OR occupation* OR physical OR psychological OR mental OR emotional ) W/3 stress ) )

40 TITLE-ABS-KEY ( ( ( work* OR job OR job-related OR occupation* OR physical OR psychological OR mental OR emotional ) W/3 anxiety ) )

41 TITLE-ABS-KEY ( ( mental W/3 ( disorder* OR illness* OR fatigue ) ) )

42 TITLE-ABS-KEY ( ( ( reduced OR declining OR deteriorating OR mental OR physical OR psychological OR emotional OR occupation* OR work-related OR work* ) W/3 health ) )

43 TITLE-ABS-KEY ( ( ( diminished OR job-related OR job OR work* OR work-related OR mental OR physical OR psychological OR occupation* OR emotional ) W/3 wellness ) )

44 TITLE-ABS-KEY ( ( ( diminished OR reduced OR deteriorating OR declining OR job-related OR job OR work* OR work-related OR mental OR physical OR psychological OR occupation* OR emotional ) W/3 well-being ) )

45 TITLE-ABS-KEY ( ( musculoskeletal W/3 ( disorder* OR condition* OR issue* OR disease* OR pain ) ) )

46 TITLE-ABS-KEY ( ( ( physical OR somatic OR bodily ) W/3 pain ) )

47 TITLE-ABS-KEY ( ( ( physical OR bodily OR somatic ) W/ discomfort ) )

48 TITLE-ABS-KEY ( ( ( physical OR bodily OR somatic ) W/3 distress ) )

49 TITLE-ABS-KEY ( "reduced performance" )

50 TITLE-ABS-KEY ( "diminished productivity" )

51 TITLE-ABS-KEY ( demotivation )

52 TITLE-ABS-KEY ( disengagement )

53 TITLE-ABS-KEY ( disinterest )

54 TITLE-ABS-KEY ( "lack of motivation" )

55 TITLE-ABS-KEY ( "change of job" )

56 TITLE-ABS-KEY ( "employment change*" )

57 TITLE-ABS-KEY ( "job transition*" )

58 TITLE-ABS-KEY ( "career shift*" )

59 TITLE-ABS-KEY ( ( ( family OR co-worker OR coworker OR work* OR workplace OR interpersonal OR peer ) W/3 conflict* ) )

60 TITLE-ABS-KEY ( ( absenteeism OR non-attendance OR "absence from work*" OR "skipping work*" OR "missing workday*" ) )

61 TITLE-ABS-KEY ( ( presenteeism OR "attending work while ill" OR "work* while sick" OR on-the-job-sickness OR "work* despite illness" ) )

62 TITLE-ABS-KEY ( ( "poor health behavio#r*" OR "unhealthy habit*" OR "negative health practice*" ) )

63 ( TITLE-ABS-KEY ( ( ( work* OR job OR job-related OR occupation* OR physical OR psychological OR mental OR emotional ) W/3 fatigue ) ) ) OR ( TITLE-ABS-KEY ( ( ( work* OR job OR job-related OR occupation* OR physical OR psychological OR mental OR emotional ) W/3 burnout ) ) ) OR ( TITLE-ABS-KEY ( ( ( work* OR job OR job-related OR occupation* OR physical OR psychological OR mental OR emotional ) W/3 exhaustion ) ) ) OR ( TITLE-ABS-KEY ( ( ( work* OR job OR job-related OR occupation* OR physical OR psychological OR mental OR emotional ) W/3 tension ) ) ) OR ( TITLE-ABS-KEY ( ( ( work* OR job OR job-related OR occupation* OR physical OR psychological OR mental OR emotional ) W/3 stress ) ) ) OR ( TITLE-ABS-KEY ( ( ( work* OR job OR job-related OR occupation* OR physical OR psychological OR mental OR emotional ) W/3 anxiety ) ) ) OR ( TITLE-ABS-KEY ( ( mental W/3 ( disorder* OR illness* OR fatigue ) ) ) ) OR ( TITLE-ABS-KEY ( ( ( reduced OR declining OR deteriorating OR mental OR physical OR psychological OR emotional OR occupation* OR work-related OR work* ) W/3 health ) ) ) OR ( TITLE-ABS-KEY ( ( ( diminished OR job-related OR job OR work* OR work-related OR mental OR physical OR psychological OR occupation* OR emotional ) W/3 wellness ) ) ) OR ( TITLE-ABS-KEY ( ( ( diminished OR reduced OR deteriorating OR declining OR job-related OR job OR work* OR work-related OR mental OR physical OR psychological OR occupation* OR emotional ) W/3 well-being ) ) ) OR ( TITLE-ABS-KEY ( ( musculoskeletal W/3 ( disorder* OR condition* OR issue* OR disease* OR pain ) ) ) ) OR ( TITLE-ABS-KEY ( ( ( physical OR somatic OR bodily ) W/3 pain ) ) ) OR ( TITLE-ABS-KEY ( ( ( physical OR bodily OR somatic ) W/ discomfort ) ) ) OR ( TITLE-ABS-KEY ( ( ( physical OR bodily OR somatic ) W/3 distress ) ) ) OR ( TITLE-ABS-KEY ( "reduced performance" ) ) OR ( TITLE-ABS-KEY ( "diminished productivity" ) ) OR ( TITLE-ABS-KEY ( demotivation ) ) OR ( TITLE-ABS-KEY ( disengagement ) ) OR ( TITLE-ABS-KEY ( disinterest ) ) OR ( TITLE-ABS-KEY ( "lack of motivation" ) ) OR ( TITLE-ABS-KEY ( "change of job" ) ) OR ( TITLE-ABS-KEY ( "employment change*" ) ) OR ( TITLE-ABS-KEY ( "job transition*" ) ) OR ( TITLE-ABS-KEY ( "career shift*" ) ) OR ( TITLE-ABS-KEY ( ( ( family OR co-worker OR coworker OR work* OR workplace OR interpersonal OR peer ) W/3 conflict* ) ) ) OR ( TITLE-ABS-KEY ( ( absenteeism OR non-attendance OR "absence from work*" OR "skipping work*" OR "missing workday*" ) ) ) OR ( TITLE-ABS-KEY ( ( presenteeism OR "attending work while ill" OR "work* while sick" OR on-the-job-sickness OR "work* despite illness" ) ) ) OR ( TITLE-ABS-KEY ( ( "poor health behavio#r*" OR "unhealthy habit*" OR "negative health practice*" ) ) )

64 ( ( TITLE-ABS-KEY ( telework* ) ) OR ( TITLE-ABS-KEY ( telecommut* ) ) OR ( TITLE-ABS-KEY ( ( telework* W/3 ( professional* OR employ* OR job OR staff* ) ) ) ) OR ( TITLE-ABS-KEY ( ( virtual* W/3 ( professional* OR work* OR employ* OR job OR staff* ) ) ) ) OR ( TITLE-ABS-KEY ( ( remote* W/3 ( professional* OR work* OR employ* OR job OR staff* ) ) ) ) OR ( TITLE-ABS-KEY ( ( off-site W/3 ( professional* OR work* OR employ* OR job OR staff* ) ) ) ) OR ( TITLE-ABS-KEY ( ( home-based W/3 ( professional* OR work* OR employ* OR job OR staff* OR telework* ) ) ) ) OR ( TITLE-ABS-KEY ( ( asynchronous W/3 ( professional* OR work* OR employ* OR job OR staff* OR telework* ) ) ) ) OR ( TITLE-ABS-KEY ( home-work* ) ) OR ( TITLE-ABS-KEY ( "work* from home" ) ) OR ( TITLE-ABS-KEY ( "work* remotely" ) ) OR ( TITLE-ABS-KEY ( ( e-work* OR ework* ) ) ) OR ( TITLE-ABS-KEY ( "flexible work*" ) ) OR ( TITLE-ABS-KEY ( "hybrid work*" ) ) ) AND ( ( TITLE-ABS-KEY ( ( work* W/3 ( "after hour*" OR "beyond hour*" OR "extended hour*" OR "outside agreed hour*" OR "past schedule*" OR "outside normal hour*" ) ) ) ) OR ( TITLE-ABS-KEY ( "long* work* hour*" ) ) OR ( TITLE-ABS-KEY ( "irregular hour*" ) ) OR ( TITLE-ABS-KEY ( "beyond work* hour*" ) ) OR ( TITLE-ABS-KEY ( leaveism* ) ) OR ( TITLE-ABS-KEY ( ( extended W/3 ( period* OR hour* OR shift* OR schedule* OR "work schedule*" ) ) ) ) OR ( TITLE-ABS-KEY ( "night work*" ) ) OR ( TITLE-ABS-KEY ( "weekend work*" ) ) OR ( TITLE-ABS-KEY ( ( ( informal OR work* ) W/3 overtime ) ) ) OR ( TITLE-ABS-KEY ( "work* late" ) ) OR ( TITLE-ABS-KEY ( "work* off the clock" ) ) OR ( TITLE-ABS-KEY ( "stay* late" ) ) OR ( TITLE-ABS-KEY ( overwork* ) ) OR ( TITLE-ABS-KEY ( workaholism ) ) OR ( TITLE-ABS-KEY ( "work-life balance" ) ) OR ( TITLE-ABS-KEY ( "work load" ) ) OR ( TITLE-ABS-KEY ( "work scheduling" ) ) OR ( TITLE-ABS-KEY ( "work rest cycle*" ) ) ) AND ( ( TITLE-ABS-KEY ( ( ( work* OR job OR job-related OR occupation* OR physical OR psychological OR mental OR emotional ) W/3 fatigue ) ) ) OR ( TITLE-ABS-KEY ( ( ( work* OR job OR job-related OR occupation* OR physical OR psychological OR mental OR emotional ) W/3 burnout ) ) ) OR ( TITLE-ABS-KEY ( ( ( work* OR job OR job-related OR occupation* OR physical OR psychological OR mental OR emotional ) W/3 exhaustion ) ) ) OR ( TITLE-ABS-KEY ( ( ( work* OR job OR job-related OR occupation* OR physical OR psychological OR mental OR emotional ) W/3 tension ) ) ) OR ( TITLE-ABS-KEY ( ( ( work* OR job OR job-related OR occupation* OR physical OR psychological OR mental OR emotional ) W/3 stress ) ) ) OR ( TITLE-ABS-KEY ( ( ( work* OR job OR job-related OR occupation* OR physical OR psychological OR mental OR emotional ) W/3 anxiety ) ) ) OR ( TITLE-ABS-KEY ( ( mental W/3 ( disorder* OR illness* OR fatigue ) ) ) ) OR ( TITLE-ABS-KEY ( ( ( reduced OR declining OR deteriorating OR mental OR physical OR psychological OR emotional OR occupation* OR work-related OR work* ) W/3 health ) ) ) OR ( TITLE-ABS-KEY ( ( ( diminished OR job-related OR job OR work* OR work-related OR mental OR physical OR psychological OR occupation* OR emotional ) W/3 wellness ) ) ) OR ( TITLE-ABS-KEY ( ( ( diminished OR reduced OR deteriorating OR declining OR job-related OR job OR work* OR work-related OR mental OR physical OR psychological OR occupation* OR emotional ) W/3 well-being ) ) ) OR ( TITLE-ABS-KEY ( ( musculoskeletal W/3 ( disorder* OR condition* OR issue* OR disease* OR pain ) ) ) ) OR ( TITLE-ABS-KEY ( ( ( physical OR somatic OR bodily ) W/3 pain ) ) ) OR ( TITLE-ABS-KEY ( ( ( physical OR bodily OR somatic ) W/ discomfort ) ) ) OR ( TITLE-ABS-KEY ( ( ( physical OR bodily OR somatic ) W/3 distress ) ) ) OR ( TITLE-ABS-KEY ( "reduced performance" ) ) OR ( TITLE-ABS-KEY ( "diminished productivity" ) ) OR ( TITLE-ABS-KEY ( demotivation ) ) OR ( TITLE-ABS-KEY ( disengagement ) ) OR ( TITLE-ABS-KEY ( disinterest ) ) OR ( TITLE-ABS-KEY ( "lack of motivation" ) ) OR ( TITLE-ABS-KEY ( "change of job" ) ) OR ( TITLE-ABS-KEY ( "employment change*" ) ) OR ( TITLE-ABS-KEY ( "job transition*" ) ) OR ( TITLE-ABS-KEY ( "career shift*" ) ) OR ( TITLE-ABS-KEY ( ( ( family OR co-worker OR coworker OR work* OR workplace OR interpersonal OR peer ) W/3 conflict* ) ) ) OR ( TITLE-ABS-KEY ( ( absenteeism OR non-attendance OR "absence from work*" OR "skipping work*" OR "missing workday*" ) ) ) OR ( TITLE-ABS-KEY ( ( presenteeism OR "attending work while ill" OR "work* while sick" OR on-the-job-sickness OR "work* despite illness" ) ) ) OR ( TITLE-ABS-KEY ( ( "poor health behavio#r*" OR "unhealthy habit*" OR "negative health practice*" ) ) ) )

65 ( ( TITLE-ABS-KEY ( telework* ) ) OR ( TITLE-ABS-KEY ( telecommut* ) ) OR ( TITLE-ABS-KEY ( ( telework* W/3 ( professional* OR employ* OR job OR staff* ) ) ) ) OR ( TITLE-ABS-KEY ( ( virtual* W/3 ( professional* OR work* OR employ* OR job OR staff* ) ) ) ) OR ( TITLE-ABS-KEY ( ( remote* W/3 ( professional* OR work* OR employ* OR job OR staff* ) ) ) ) OR ( TITLE-ABS-KEY ( ( off-site W/3 ( professional* OR work* OR employ* OR job OR staff* ) ) ) ) OR ( TITLE-ABS-KEY ( ( home-based W/3 ( professional* OR work* OR employ* OR job OR staff* OR telework* ) ) ) ) OR ( TITLE-ABS-KEY ( ( asynchronous W/3 ( professional* OR work* OR employ* OR job OR staff* OR telework* ) ) ) ) OR ( TITLE-ABS-KEY ( home-work* ) ) OR ( TITLE-ABS-KEY ( "work* from home" ) ) OR ( TITLE-ABS-KEY ( "work* remotely" ) ) OR ( TITLE-ABS-KEY ( ( e-work* OR ework* ) ) ) OR ( TITLE-ABS-KEY ( "flexible work*" ) ) OR ( TITLE-ABS-KEY ( "hybrid work*" ) ) ) AND ( ( TITLE-ABS-KEY ( ( work* W/3 ( "after hour*" OR "beyond hour*" OR "extended hour*" OR "outside agreed hour*" OR "past schedule*" OR "outside normal hour*" ) ) ) ) OR ( TITLE-ABS-KEY ( "long* work* hour*" ) ) OR ( TITLE-ABS-KEY ( "irregular hour*" ) ) OR ( TITLE-ABS-KEY ( "beyond work* hour*" ) ) OR ( TITLE-ABS-KEY ( leaveism* ) ) OR ( TITLE-ABS-KEY ( ( extended W/3 ( period* OR hour* OR shift* OR schedule* OR "work schedule*" ) ) ) ) OR ( TITLE-ABS-KEY ( "night work*" ) ) OR ( TITLE-ABS-KEY ( "weekend work*" ) ) OR ( TITLE-ABS-KEY ( ( ( informal OR work* ) W/3 overtime ) ) ) OR ( TITLE-ABS-KEY ( "work* late" ) ) OR ( TITLE-ABS-KEY ( "work* off the clock" ) ) OR ( TITLE-ABS-KEY ( "stay* late" ) ) OR ( TITLE-ABS-KEY ( overwork* ) ) OR ( TITLE-ABS-KEY ( workaholism ) ) OR ( TITLE-ABS-KEY ( "work-life balance" ) ) OR ( TITLE-ABS-KEY ( "work load" ) ) OR ( TITLE-ABS-KEY ( "work scheduling" ) ) OR ( TITLE-ABS-KEY ( "work rest cycle*" ) ) ) AND ( ( TITLE-ABS-KEY ( ( ( work* OR job OR job-related OR occupation* OR physical OR psychological OR mental OR emotional ) W/3 fatigue ) ) ) OR ( TITLE-ABS-KEY ( ( ( work* OR job OR job-related OR occupation* OR physical OR psychological OR mental OR emotional ) W/3 burnout ) ) ) OR ( TITLE-ABS-KEY ( ( ( work* OR job OR job-related OR occupation* OR physical OR psychological OR mental OR emotional ) W/3 exhaustion ) ) ) OR ( TITLE-ABS-KEY ( ( ( work* OR job OR job-related OR occupation* OR physical OR psychological OR mental OR emotional ) W/3 tension ) ) ) OR ( TITLE-ABS-KEY ( ( ( work* OR job OR job-related OR occupation* OR physical OR psychological OR mental OR emotional ) W/3 stress ) ) ) OR ( TITLE-ABS-KEY ( ( ( work* OR job OR job-related OR occupation* OR physical OR psychological OR mental OR emotional ) W/3 anxiety ) ) ) OR ( TITLE-ABS-KEY ( ( mental W/3 ( disorder* OR illness* OR fatigue ) ) ) ) OR ( TITLE-ABS-KEY ( ( ( reduced OR declining OR deteriorating OR mental OR physical OR psychological OR emotional OR occupation* OR work-related OR work* ) W/3 health ) ) ) OR ( TITLE-ABS-KEY ( ( ( diminished OR job-related OR job OR work* OR work-related OR mental OR physical OR psychological OR occupation* OR emotional ) W/3 wellness ) ) ) OR ( TITLE-ABS-KEY ( ( ( diminished OR reduced OR deteriorating OR declining OR job-related OR job OR work* OR work-related OR mental OR physical OR psychological OR occupation* OR emotional ) W/3 well-being ) ) ) OR ( TITLE-ABS-KEY ( ( musculoskeletal W/3 ( disorder* OR condition* OR issue* OR disease* OR pain ) ) ) ) OR ( TITLE-ABS-KEY ( ( ( physical OR somatic OR bodily ) W/3 pain ) ) ) OR ( TITLE-ABS-KEY ( ( ( physical OR bodily OR somatic ) W/ discomfort ) ) ) OR ( TITLE-ABS-KEY ( ( ( physical OR bodily OR somatic ) W/3 distress ) ) ) OR ( TITLE-ABS-KEY ( "reduced performance" ) ) OR ( TITLE-ABS-KEY ( "diminished productivity" ) ) OR ( TITLE-ABS-KEY ( demotivation ) ) OR ( TITLE-ABS-KEY ( disengagement ) ) OR ( TITLE-ABS-KEY ( disinterest ) ) OR ( TITLE-ABS-KEY ( "lack of motivation" ) ) OR ( TITLE-ABS-KEY ( "change of job" ) ) OR ( TITLE-ABS-KEY ( "employment change*" ) ) OR ( TITLE-ABS-KEY ( "job transition*" ) ) OR ( TITLE-ABS-KEY ( "career shift*" ) ) OR ( TITLE-ABS-KEY ( ( ( family OR co-worker OR coworker OR work* OR workplace OR interpersonal OR peer ) W/3 conflict* ) ) ) OR ( TITLE-ABS-KEY ( ( absenteeism OR non-attendance OR "absence from work*" OR "skipping work*" OR "missing workday*" ) ) ) OR ( TITLE-ABS-KEY ( ( presenteeism OR "attending work while ill" OR "work* while sick" OR on-the-job-sickness OR "work* despite illness" ) ) ) OR ( TITLE-ABS-KEY ( ( "poor health behavio#r*" OR "unhealthy habit*" OR "negative health practice*" ) ) ) ) AND PUBYEAR > 2009 AND PUBYEAR < 2025 AND ( LIMIT-TO ( LANGUAGE , "english" ) )

***Business Source Premier (Ebsco)***

1 DE "TELECOMMUTING" OR DE "TELECONFERENCING"

2 TI telework* OR AB telework*

3 TI telecommut* OR AB telecommut*

4 TI ( (telework* N3 (professional* or employ* or job or staff*)) ) OR AB ( (telework* N3 (professional* or employ* or job or staff*)) )

5 TI ( (virtual* N3 (professional* or work* or employ* or job or staff*)) ) OR AB ( (virtual* N3 (professional* or work* or employ* or job or staff*)) )

6 TI ( (remote* N3 (professional* or work* or employ* or job or staff*)) ) OR AB ( (remote* N3 (professional* or work* or employ* or job or staff*)) )

7 TI ( (off-site N3 (professional* or work* or employ* or job or staff*)) ) OR AB ( (off-site N3 (professional* or work* or employ* or job or staff*)) )

8 TI ( (home-based N3 (professional* or work* or employ* or job or staff* or telework*)) ) OR AB ( (home-based N3 (professional* or work* or employ* or job or staff* or telework*)) )

9 TI home-work* OR AB home-work*

10 TI "work* from home" OR AB "work* from home"

11 TI ( (asynchronous N3 (professional* or work* or employ* or job or staff* or telework*)) ) OR AB ( (asynchronous N3 (professional* or work* or employ* or job or staff* or telework*)) )

12 TI "work* remotely" OR AB "work* remotely"

13 TI ( (e-work* or ework*) ) OR AB ( (e-work* or ework*) )

14 TI "flexible work*" OR AB "flexible work*"

15 TI "hybrid work*" OR AB "hybrid work*"

16 S1 OR S2 OR S3 OR S4 OR S5 OR S6 OR S7 OR S8 OR S9 OR S10 OR S11 OR S12 OR S13 OR S14 OR S15

17 (DE "WORKAHOLISM") OR (DE "WORK-life balance")

18 TI ( (work* N3 ("after hour*" or "beyond hour*" or "extended hour*" or "outside agreed hour*" or "past schedule*" or "outside normal hour*")) ) OR AB ( (work* N3 ("after hour*" or "beyond hour*" or "extended hour*" or "outside agreed hour*" or "past schedule*" or "outside normal hour*")) )

19 TI "long* work* hour*" OR AB "long* work* hour*"

20 TI "irregular hour*" OR AB "irregular hour*"

21 TI "beyond work* hour*" OR AB "beyond work* hour*"

22 TI leaveism* OR AB leaveism*

23 TI ( (extended N3 (period* or hour* or shift* or schedule* or "work schedule*")) ) OR AB ( (extended N3 (period* or hour* or shift* or schedule* or "work schedule*")) )

24 TI "night work*" OR AB "night work*" OR TI "night shift*" OR AB "night shift*"

25 TI "weekend work*" OR AB "weekend work*"

26 TI ( ((informal or work*) N3 overtime) ) OR AB ( ((informal or work*) N3 overtime) )

27 TI "work* late" OR AB "work* late"

28 TI "work* off the clock" OR AB "work* off the clock"

29 TI "staying late" OR AB "staying late"

30 TI overwork* OR AB overwork*

31 S17 OR S18 OR S19 OR S20 OR S21 OR S22 OR S23 OR S24 OR S25 OR S26 OR S27 OR S28 OR S29 OR S30

32 TI ( ((work* or job or job-related or occupation* or physical or psychological or mental or emotional) N3 fatigue) ) OR AB ( ((work* or job or job-related or occupation* or physical or psychological or mental or emotional) N3 fatigue) )

33 DE "PSYCHOLOGICAL burnout"

34 TI ( ((work* or job or job-related or occupation* or physical or psychological or mental or emotional) N3 burnout) ) OR AB ( ((work* or job or job-related or occupation* or physical or psychological or mental or emotional) N3 burnout) )

35 TI ( ((work* or job or job-related or occupation* or physical or psychological or mental or emotional) N3 exhaustion) ) OR AB ( ((work* or job or job-related or occupation* or physical or psychological or mental or emotional) N3 exhaustion) )

36 TI ( ((work* or job or job-related or occupation* or physical or psychological or mental or emotional) N3 tension) ) OR AB ( ((work* or job or job-related or occupation* or physical or psychological or mental or emotional) N3 tension) )

37 DE "JOB stress"

38 TI ( ((work* or job or job-related or occupation* or physical or psychological or mental or emotional) N3 stress) ) OR AB ( ((work* or job or job-related or occupation* or physical or psychological or mental or emotional) N3 stress) )

39 TI ( ((work* or job or job-related or occupation* or physical or psychological or mental or emotional) N3 anxiety) ) OR AB ( ((work* or job or job-related or occupation* or physical or psychological or mental or emotional) N3 anxiety) )

40 TI ( (mental N3 (disorder* or illness* or fatigue)) ) OR AB ( (mental N3 (disorder* or illness* or fatigue)) )

41 TI ( ((reduced or declining or deteriorating or mental or physical or psychological or emotional or occupation* or work-related or work*) N3 health) ) OR AB ( ((reduced or declining or deteriorating or mental or physical or psychological or emotional or occupation* or work-related or work*) N3 health) )

42 TI ( ((diminished or job-related or job or work* or work-related or mental or physical or psychological or occupation* or emotional) N3 wellness) ) OR AB ( ((diminished or job-related or job or work* or work-related or mental or physical or psychological or occupation* or emotional) N3 wellness) )

43 DE "JOB satisfaction"

44 TI ( ((diminished or reduced or deteriorating or declining or job-related or job or work* or work-related or mental or physical or psychological or occupation* or emotional) N3 well-being) ) OR AB ( ((diminished or reduced or deteriorating or declining or job-related or job or work* or work-related or mental or physical or psychological or occupation* or emotional) N3 well-being) )

45 TI ( (musculoskeletal N3 (disorder* or condition* or issue* or disease* or pain)) ) OR AB ( (musculoskeletal N3 (disorder* or condition* or issue* or disease* or pain)) )

46 TI ( ((physical or somatic or bodily) N3 pain) ) OR AB ( ((physical or somatic or bodily) N3 pain) )

47 TI ( ((physical or bodily or somatic) N3 discomfort) ) OR AB ( ((physical or bodily or somatic) N3 discomfort) )

48 TI ( ((physical or bodily or somatic) N3 distress) ) OR AB ( ((physical or bodily or somatic) N3 distress) )

49 TI "reduced performance" OR AB "reduced performance"

50 TI "diminished productivity" OR AB "diminished productivity"

51 TI demotivation OR AB demotivation

52 TI disengagement OR AB disengagement

53 TI disinterest OR AB disinterest

54 TI "lack of motivation" OR AB "lack of motivation"

55 TI "change of job" OR AB "change of job" OR TI "change of position" OR AB "change of position"

56 TI "employment change*" OR AB "employment change*"

57 DE "JOB security"

58 TI "job transition*" OR AB "job transition*"

59 TI "career shift*" OR AB "career shift*"

60 TI ( ((family or co-worker or coworker or work* or workplace or interpersonal or peer) N3 conflict*) ) OR AB ( ((family or co-worker or coworker or work* or workplace or interpersonal or peer) N3 conflict*) )

61 DE "PRESENTEEISM (Labor)"

62 TI ( (presenteeism or "attending work while ill" or "work* while sick" or on-the-job-sickness or "working despite illness") ) OR AB ( (presenteeism or "attending work while ill" or "work* while sick" or on-the-job-sickness or "working despite illness") )

63 TI ( (absenteeism or non-attendance or "absence from work*" or "skipping work*" or "missing workday*") ) OR AB ( (absenteeism or non-attendance or "absence from work*" or "skipping work*" or "missing workday*") )

64 TI ( ("poor health behavio?r*" or "unhealthy habit*" or "negative health practice*") ) OR AB ( ("poor health behavio?r*" or "unhealthy habit*" or "negative health practice*") )

65 S32 OR S33 OR S34 OR S35 OR S36 OR S37 OR S38 OR S39 OR S40 OR S41 OR S42 OR S43 OR S44 OR S45 OR S46 OR S47 OR S48 OR S49 OR S50 OR S51 OR S52 OR S53 OR S54 OR S55 OR S56 OR S57 OR S58 OR S59 OR S60 OR S61 OR S62 OR S63 OR S64

67 S16 AND S31 AND S65

68 S16 AND S31 AND S65 (limit to english language and yr="2010 -Current")

***CINAHL (Ebsco)***

1 (MH "Telecommuting")

2 (MH "Teleconferencing") OR (MH "Videoconferencing")

3 TI telework* OR AB telework*

4 TI telecommut* OR AB telecommut*

5 TI ( (telework* N3 (professional* or employ* or job or staff*)) ) OR AB ( (telework* N3 (professional* or employ* or job or staff*)) )

6 TI ( (virtual* N3 (professional* or work* or employ* or job or staff*)) ) OR AB ( (virtual* N3 (professional* or work* or employ* or job or staff*)) )

7 TI ( (remote* N3 (professional* or work* or employ* or job or staff*)) ) OR AB ( (remote* N3 (professional* or work* or employ* or job or staff*)) )

8 TI ( (off-site N3 (professional* or work* or employ* or job or staff*)) ) OR AB ( (off-site N3 (professional* or work* or employ* or job or staff*)) )

9 TI ( (home-based N3 (professional* or work* or employ* or job or staff* or telework*)) ) OR AB ( (home-based N3 (professional* or work* or employ* or job or staff* or telework*)) )

10 TI home-work* OR AB home-work*

11 TI "work* from home" OR AB "work* from home"

12 TI ( (asynchronous N3 (professional* or work* or employ* or job or staff* or telework*)) ) OR AB ( (asynchronous N3 (professional* or work* or employ* or job or staff* or telework*)) )

13 TI "work* remotely" OR AB "work* remotely"

14 TI ( (e-work* or ework*) ) OR AB ( (e-work* or ework*) )

15 TI "flexible work*" OR AB "flexible work*"

16 TI "hybrid work*" OR AB "hybrid work*"

17 S1 OR S2 OR S3 OR S4 OR S5 OR S6 OR S7 OR S8 OR S9 OR S10 OR S11 OR S12 OR S13 OR S14 OR S15 OR S16

18 (MH "Work-Life Balance") OR (MH "Quality of Working Life") OR (MH "Work Engagement")

19 TI ( (work* N3 ("after hour*" or "beyond hour*" or "extended hour*" or "outside agreed hour*" or "past schedule*" or "outside normal hour*")) ) OR AB ( (work* N3 ("after hour*" or "beyond hour*" or "extended hour*" or "outside agreed hour*" or "past schedule*" or "outside normal hour*")) )

20 TI "long* work* hour*" OR AB "long* work* hour*"

21 TI "irregular hour*" OR AB "irregular hour*"

22 TI "beyond work* hour*" OR AB "beyond work* hour*"

23 TI leaveism* OR AB leaveism*

24 TI ( (extended N3 (period* or hour* or shift* or schedule* or "work schedule*")) ) OR AB ( (extended N3 (period* or hour* or shift* or schedule* or "work schedule*")) )

25 TI "night work*" OR AB "night work*" OR TI "night shift*" OR AB "night shift*"

26 TI "weekend work*" OR AB "weekend work*"

27 TI ( ((informal or work*) N3 overtime) ) OR AB ( ((informal or work*) N3 overtime) )

28 TI "work* late" OR AB "work* late"

29 TI "work* off the clock" OR AB "work* off the clock"

30 TI "staying late" OR AB "staying late"

31 TI overwork* OR AB overwork*

32 S18 OR S19 OR S20 OR S21 OR S22 OR S23 OR S24 OR S25 OR S26 OR S27 OR S28 OR S29 OR S30 OR S31

33 TI ( ((work* or job or job-related or occupation* or physical or psychological or mental or emotional) N3 fatigue) ) OR AB ( ((work* or job or job-related or occupation* or physical or psychological or mental or emotional) N3 fatigue) )

34 (MH "Burnout, Professional")

35 TI ( ((work* or job or job-related or occupation* or physical or psychological or mental or emotional) N3 burnout) ) OR AB ( ((work* or job or job-related or occupation* or physical or psychological or mental or emotional) N3 burnout) )

36 TI ( ((work* or job or job-related or occupation* or physical or psychological or mental or emotional) N3 exhaustion) ) OR AB ( ((work* or job or job-related or occupation* or physical or psychological or mental or emotional) N3 exhaustion) )

37 TI ( ((work* or job or job-related or occupation* or physical or psychological or mental or emotional) N3 tension) ) OR AB ( ((work* or job or job-related or occupation* or physical or psychological or mental or emotional) N3 tension) )

38 (MH "Stress, Occupational")

39 TI ( ((work* or job or job-related or occupation* or physical or psychological or mental or emotional) N3 stress) ) OR AB ( ((work* or job or job-related or occupation* or physical or psychological or mental or emotional) N3 stress) )

40 TI ( ((work* or job or job-related or occupation* or physical or psychological or mental or emotional) N3 anxiety) ) OR AB ( ((work* or job or job-related or occupation* or physical or psychological or mental or emotional) N3 anxiety) )

41 TI ( (mental N3 (disorder* or illness* or fatigue)) ) OR AB ( (mental N3 (disorder* or illness* or fatigue)) )

42 TI ( ((reduced or declining or deteriorating or mental or physical or psychological or emotional or occupation* or work-related or work*) N3 health) ) OR AB ( ((reduced or declining or deteriorating or mental or physical or psychological or emotional or occupation* or work-related or work*) N3 health) )

43 TI ( ((diminished or job-related or job or work* or work-related or mental or physical or psychological or occupation* or emotional) N3 wellness) ) OR AB ( ((diminished or job-related or job or work* or work-related or mental or physical or psychological or occupation* or emotional) N3 wellness) )

44 (MH "Job Satisfaction")

45 TI ( ((diminished or reduced or deteriorating or declining or job-related or job or work* or work-related or mental or physical or psychological or occupation* or emotional) N3 well-being) ) OR AB ( ((diminished or reduced or deteriorating or declining or job-related or job or work* or work-related or mental or physical or psychological or occupation* or emotional) N3 well-being) )

46 TI ( (musculoskeletal N3 (disorder* or condition* or issue* or disease* or pain)) ) OR AB ( (musculoskeletal N3 (disorder* or condition* or issue* or disease* or pain)) )

47 TI ( ((physical or somatic or bodily) N3 pain) ) OR AB ( ((physical or somatic or bodily) N3 pain) )

48 TI ( ((physical or bodily or somatic) N3 discomfort) ) OR AB ( ((physical or bodily or somatic) N3 discomfort) )

49 TI ( ((physical or bodily or somatic) N3 distress) ) OR AB ( ((physical or bodily or somatic) N3 distress) )

50 TI "reduced performance" OR AB "reduced performance"

51 TI "diminished productivity" OR AB "diminished productivity"

52 TI demotivation OR AB demotivation

53 TI disengagement OR AB disengagement

54 TI disinterest OR AB disinterest

55 TI "lack of motivation" OR AB "lack of motivation"

56 TI "change of job" OR AB "change of job" OR TI "change of position" OR AB "change of position"

57 TI "employment change*" OR AB "employment change*"

58 (MH "Job Security")

59 TI "job transition*" OR AB "job transition*"

60 TI "career shift*" OR AB "career shift*"

61 TI ( ((family or co-worker or coworker or work* or workplace or interpersonal or peer) N3 conflict*) ) OR AB ( ((family or co-worker or coworker or work* or workplace or interpersonal or peer) N3 conflict*) )

62 (MH "Presenteeism")

63 TI ( (presenteeism or "attending work while ill" or "work* while sick" or on-the-job-sickness or "working despite illness") ) OR AB ( (presenteeism or "attending work while ill" or "work* while sick" or on-the-job-sickness or "working despite illness") )

64 (MH "Absenteeism")

65 TI ( (absenteeism or non-attendance or "absence from work*" or "skipping work*" or "missing workday*") ) OR AB ( (absenteeism or non-attendance or "absence from work*" or "skipping work*" or "missing workday*") )

66 TI ( ("poor health behavio?r*" or "unhealthy habit*" or "negative health practice*") ) OR AB ( ("poor health behavio?r*" or "unhealthy habit*" or "negative health practice*") )

67 S33 OR S34 OR S35 OR S36 OR S37 OR S38 OR S39 OR S40 OR S41 OR S42 OR S43 OR S44 OR S45 OR S46 OR S47 OR S48 OR S49 OR S50 OR S51 OR S52 OR S53 OR S54 OR S55 OR S56 OR S57 OR S58 OR S59 OR S60 OR S61 OR S62 OR S63 OR S64 OR S65 OR S66

68 S17 AND S32 AND S67

69 S17 AND S32 AND S67 (limit to english language and yr="2010 -Current")

***Sociological Abstracts (ProQuest)***

1 MAINSUBJECT.EXACT.EXPLODE("Telecommuting")

2 noft(telework*)

3 noft(telecommut*)

4 noft((telework* NEAR/3 (professional* or employ* or job or staff*)))

5 noft((virtual* NEAR/3 (professional* or work* or employ* or job or staff*)))

6 noft((remote* NEAR/3 (professional* or work* or employ* or job or staff*)))

7 noft((off-site NEAR/3 (professional* or work* or employ* or job or staff*)))

8 noft((home-based NEAR/3 (professional* or work* or employ* or job or staff* or telework*)))

9 noft((asynchronous NEAR/3 (professional* or work* or employ* or job or staff* or telework*)))

10 noft(home-work*)

11 noft("work* from home")

12 noft("work* remotely")

13 noft((e-work* or ework*))

14 noft("flexible work*")

15 noft("hybrid work*")

16 MAINSUBJECT.EXACT("Work at home")

17 [S1] OR [S2] OR [S3] OR [S4] OR [S5] OR [S6] OR [S7] OR [S8] OR [S9] OR [S10] OR [S11] OR [S12] OR [S13] OR [S14] OR [S15] OR [S16]

18 MAINSUBJECT.EXACT("Occupational stress") OR MAINSUBJECT.EXACT("Work ethic") OR MAINSUBJECT.EXACT("Worker control") OR MAINSUBJECT.EXACT("Working hours") OR MAINSUBJECT.EXACT("Work leisure relationship") OR MAINSUBJECT.EXACT("Working conditions")

19 noft((work* NEAR/3 ("after hour*" or "beyond hour*" or "extended hour*" or "outside agreed hour*" or "past schedule*" or "outside normal hour*")))

20 noft("long* work* hour*")

21 noft("irregular hour*")

22 noft("beyond work* hour*")

23 noft("leaveism*")

24 noft((extended NEAR/3 (period* or hour* or shift* or schedule* or "work schedule*")))

25 noft("night work*")

26 noft("weekend work*")

27 noft(((informal or work*) NEAR/3 overtime))

28 noft("work* late")

29 noft("work* off the clock")

30 noft("stay* late")

31 noft("overwork*")

32 [S18] OR [S19] OR [S20] OR [S21] OR [S22] OR [S23] OR [S24] OR [S25] OR [S26] OR [S27] OR [S28] OR [S29] OR [S30] OR [S31]

33 MAINSUBJECT.EXACT("Chronic fatigue syndrome") OR MAINSUBJECT.EXACT("Fatigue") OR MAINSUBJECT.EXACT("Burnout")

34 noft(((work* or job or job-related or occupation* or physical or psychological or mental or emotional) NEAR/3 fatigue))

35 noft(((work* or job or job-related or occupation* or physical or psychological or mental or emotional) NEAR/3 burnout))

36 noft(((work* or job or job-related or occupation* or physical or psychological or mental or emotional) NEAR/3 exhaustion))

37 noft(((work* or job or job-related or occupation* or physical or psychological or mental or emotional) NEAR/3 tension))

38 MAINSUBJECT.EXACT("Stress") OR MAINSUBJECT.EXACT("Psychological distress") OR MAINSUBJECT.EXACT("Post traumatic stress disorder") OR MAINSUBJECT.EXACT("Emotional distress")

39 MAINSUBJECT.EXACT("Job satisfaction") OR MAINSUBJECT.EXACT("Job performance") OR MAINSUBJECT.EXACT("Job requirements")

40 noft(((work* or job or job-related or occupation* or physical or psychological or mental or emotional) NEAR/3 stress))

41 MAINSUBJECT.EXACT("Anxiety")

42 noft(((work* or job or job-related or occupation* or physical or psychological or mental or emotional) NEAR/3 anxiety))

43 MAINSUBJECT.EXACT("Mental disorders")

44 noft((mental NEAR/3 (disorder* or illness* or fatigue)))

45 noft(((reduced or declining or deteriorating or mental or physical or psychological or emotional or occupation* or work-related or work*) NEAR/3 health))

46 noft(((diminished or job-related or job or work* or work-related or mental or physical or psychological or occupation* or emotional) NEAR/3 wellness))

47 MAINSUBJECT.EXACT("Employee attitude") OR MAINSUBJECT.EXACT("Employee turnover")

48 noft(((diminished or reduced or deteriorating or declining or job-related or job or work* or work-related or mental or physical or psychological or occupation* or emotional) NEAR/3 well-being))

49 noft((musculoskeletal NEAR/3 (disorder* or condition* or issue* or disease* or pain)))

50 MAINSUBJECT.EXACT("Pain")

51 MAINSUBJECT.EXACT("Physical disabilities")

52 noft(((physical or somatic or bodily) NEAR/3 pain)) OR noft(((physical or bodily or somatic) NEAR/3 discomfort)) OR noft(((physical or bodily or somatic) NEAR/3 distress))

53 noft("reduced performance")

54 noft("diminished productivity")

55 noft(demotivation)

56 noft(disengagement)

57 noft(disinterest)

58 noft("lack of motivation")

59 noft("change of job")

60 noft("employment change*")

61 noft("job transition*")

62 noft("career shift*")

63 MAINSUBJECT.EXACT("Marital separation") OR MAINSUBJECT.EXACT("Marital disruption") OR MAINSUBJECT.EXACT("Domestic violence") OR MAINSUBJECT.EXACT("Family conflict")

64 noft(((family or co-worker or coworker or work* or workplace or interpersonal or peer) NEAR/3 conflict*))

65 MAINSUBJECT.EXACT("Absenteeism")

66 noft((absenteeism or non-attendance or "absence from work*" or "skipping work*" or "missing workday*"))

67 MAINSUBJECT.EXACT("Illness behavior")

68 noft((presenteeism or "attending work while ill" or "work* while sick" or on-the-job-sickness or "working despite illness"))

69 noft(("poor health behavio?r*" or "unhealthy habit*" or "negative health practice*"))

70 [S33] OR [S34] OR [S35] OR [S36] OR [S37] OR [S38] OR [S39] OR [S40] OR [S41] OR [S42] OR [S43] OR [S44] OR [S45] OR [S46] OR [S47] OR [S48] OR [S49] OR [S50] OR [S51] OR [S52] OR [S53] OR [S54] OR [S55] OR [S56] OR [S57] OR [S58] OR [S59] OR [S60] OR [S61] OR [S62] OR [S63] OR [S64] OR [S65] OR [S66] OR [S67] OR [S68] OR [S69]

71 [S17] AND [S32] AND [S70]

72 [S17] AND [S32] AND [S70] (limit to english language and yr="2010 -Current")
